# Evaluation of Antioxidant, Antidiabetic and Anticholinesterase Activities of *Smallanthus sonchifolius* Landraces and Correlation with Their Phytochemical Profiles

**DOI:** 10.3390/ijms160817696

**Published:** 2015-07-31

**Authors:** Daniela Russo, Patrícia Valentão, Paula B. Andrade, Eloy C. Fernandez, Luigi Milella

**Affiliations:** 1 Department of Science, Basilicata University, 85100 Potenza, Italy; 2 Rede de Química e Tecnologia/Laboratório Associado para a Química Verde (REQUIMTE/LAQV), Laboratório de Farmacognosia, Departamento de Química, Faculdade de Farmácia, Universidade do Porto, 4050-313 Porto, Portugal; E-Mails: valentao@ff.up.pt (P.V.); pandrade@ff.up.pt (P.B.A.); 3 Department of Crop Sciences and Agroforestry, Faculty of Tropical AgriSciences, Czech University of Life Sciences, 165 21 Prague, Czech Republic; E-Mail: fernandez@ftz.czu.cz

**Keywords:** *Smallanthus sonchifolius*, phytochemical profile, antioxidant activity, anti-cholinesterase activity, anti-diabetic activity

## Abstract

The present study aimed to investigate the phytochemical profile of leaf methanol extracts of fourteen *Smallanthus sonchifolius* (yacon) landraces and their antioxidant, anticholinesterase and antidiabetic activities that could lead to the finding of more effective agents for the treatment and management of Alzheimer’s disease and diabetes. For this purpose, antioxidant activity was assessed using different tests: ferric reducing ability power (FRAP), 2,2-diphenyl-1-picryl hydrazyl (DPPH), nitric oxide (˙NO) and superoxide (O_2_˙^−^) scavenging and lipid peroxidation inhibition assays. Anticholinesterase activity was investigated by quantifying the acetylcholinesterase (AChE) and butyrylcholinesterase (BChE) inhibitory activities, whereas antidiabetic activity was investigated by α-amylase and α-glucosidase inhibition tests. To understand the contribution of metabolites, phytochemical screening was also performed by high performance liquid chromatography-diode array detector (HPLC-DAD) system. Among all, methanol extract of PER09, PER04 and ECU44 landraces exhibited the highest relative antioxidant capacity index (RACI). ECU44 was found to be rich in 4,5-di-*O*-caffeoylquinic acid (CQA) and 3,5-di-*O*-CQA and displayed a good α-amylase and α-glucosidase inhibition, showing the lowest IC_50_ values. Flavonoids, instead, seem to be involved in the AChE and BChE inhibition. The results of this study revealed that the bioactive compound content differences could be determinant for the medicinal properties of this plant especially for antioxidant and antidiabetic activities.

## 1. Introduction

Medicinal plants, as source of remedies, are widely used as alternative therapeutic tools for the prevention or treatment of many diseases. Recently, great attention has been devoted to the use of natural compounds, due to their nutritional and pharmacological characteristics.

*Smallanthus sonchifolius* (Poepp. & Hendl.) H. Robinson, commonly called yacon, belongs to the *Asteraceae* family. It is a tuberous plant native to the Andes, but it has been introduced in Japan, New Zealand, Europe (primarily in Czech Republic) and United States [1]. Yacon is a perennial herb with large dark green leaves and two types of underground portions: edible tuberous roots, used by the plant for food storage, and fibrous roots used for vegetative reproduction. Each plant produces 4 to 20 edible tubers that can reach 20 kg [2]. Andean indigenous people have always used yacon as an important crop: they have used it not only for its edible tuberous roots, but also as a vegetable and medicinal plant. Roots of yacon can contain high amounts (40%–70% dry weight) of fructooligosaccharides [3,4] that are not metabolized in the human digestive tract and hence their consumption does not enhance the level of glucose in the blood. In addition, studies have reported that extracts of leaves reduce glycemia in the plasma of diabetic rats [5] and some constituents of yacon leaves inhibit the α-glucosidase enzyme involved in the diabetes [6]. For this reason yacon is considered to be a food and a remedy with a high potential for diabetics. The low energy value of its tuberous roots makes yacon an ideal foodstuff for people suffering from obesity. Leaves are also a rich source of antioxidants [7] and several studies showed that this part of the plant possesses different biological effects, like inhibition of migration of polymorphonuclear leucocytes [8], immunomodulation [9], antioxidant, cytoprotection effects [10,11,12] and antimicrobical activity [13].

The search for the bioactive compounds from medicinal plants is always an alternative mean of finding new drugs. Phytochemical screening of *S. sonchifolius* will lead to the rationalization of the use of this plant in various diseases and could also lead to the finding of more effective agents that have effective treatment roles against specific diseases. Alzheimer’s disease (AD) was firstly described in 1906 by Alois Alzheimer, a Bavarian neuropsychiatrist [14]. AD is a progressive neurodegenerative disorder of the elderly, characterized by widespread loss of central cholinergic function. The only symptomatic treatment proven effective to date is the use of cholinesterase (ChE) inhibitors to augment surviving cholinergic activity. ChE inhibitors act on the enzymes that hydrolyze acetylcholine (ACh) following synaptic release. In the healthy brain, acetylcholinesterase (AChE) predominates (80%) and butyrylcholinesterase (BChE) is considered to play a minor role in regulating brain ACh levels. Therefore both enzymes are likely to have involvement in regulating ACh levels and represent legitimate therapeutic targets to ameliorate the cholinergic deficit. Many plants have been studied by different approaches for the identification of new AChE inhibitors (AChE-Is) and different classes of plant-derived natural products have been considered as new AChE-Is potentially useful for AD treatment. Both non-alkaloids and alkaloid-derivative compounds [15] have been studied.

The incidence of diabetes mellitus in the world is increasing at an alarming rate, affecting close to 5% of its population and it is strictly related to several other diseases [16,17]. Diabetes mellitus is a complex disease characterized by gross derangement in carbohydrate, fat and protein metabolism, due to deficiency in insulin secretion and/or action [18]. Mammalian α-amylase is a prominent enzyme in the pancreatic juice, breaking down large and insoluble starch molecules into absorbable molecules, ultimately maltose [19]. α-Glucosidase, on the other hand, anchored in the mucosal brush border of the small intestine, catalyzes the end step of digestion of starch and disaccharides that are abundant in human diet [20]. Inhibitors of α-amylase and α-glucosidase delay the breakdown of carbohydrate in the small intestine and decrease the postprandial blood glucose excursion levels in diabetic patients. The inhibition of these two prominent enzymes has been found as a useful and effective strategy to lower the levels of postprandial hyperglycemia [21].

Moreover oxidative stress has been implicated in various pathological conditions involving cardiovascular disease, cancer, neurological disorders (Alzheimer’s disease and Parkinson’s disease), diabetes, ischemia/reperfusion, other diseases and ageing. Convincing evidence for the association of oxidative/nitrosative stress and acute and chronic diseases lies on validated biomarkers of oxidative stress [22]. For this reasons we have also investigated the antioxidant potential of yacon extracts by five different assays. In conclusion, the aim of this study was to investigate the biological properties, including antioxidant, antidiabetic and anticholinesterase activity, and the phytochemical profiles of leaf methanol extracts of fourteen yacon landraces.

## 2. Results and Discussion

### 2.1. Extraction Yields, Total Polyphenolic, Tannin and Flavonoid Content

Dried foliar tissue of 14 landraces of yacon was extracted by maceration technique by solvents of crescent polarity: *n*-hexane (E), chloroform (C), chloroform:methanol 9:1 (CM) and methanol (M). Dried extract yield was calculated. Data showed the different extractive capacities of each solvent. Methanol was the solvent with the highest extractive capacity, the percentage of yield ranged from 4.5% to 9.8% followed by *n*-hexane (3.2% to 6.9%); in fact, the latter solvent allows to extract mainly the chlorophylls, abundant in the leaf tissue. The percentage of yield obtained by chloroform and chloroform:methanol mixture (9:1) ranged from 2.3% to 3.8% and from 0.7% to 3.0%, respectively.

The total polyphenolic content (TPC) of different extracts of 14 genotypes of yacon was determined by Folin–Ciocalteu assay. According to literature, methanol extracts show higher content of phenolic compounds than other extracts (data not shown); phenols are also the main responsible compounds of the antioxidant activity; for this reason, the leaf methanol extracts of fourteen yacon landraces have been further analyzed [23]. Results of methanol extracts were expressed as mg of gallic acid equivalent (GAE)/g of dried extract (Table 1), by using a standard curve. Yacon landraces displayed quantitative differences in TPC, with a mean value of 74.47 mg GAE/g. The TPC ranged from 58.49 ± 1.03 to 91.07 ± 4.87 mg GAE/g of dried extract, in methanol extracts of PER07 (PER07-M) and PER09-M, respectively. Methanol extract of polyploid sample ECU44 showed a phenolic content similar to that of the mother plant (ECU41).

**Table 1:** Results of total polyphenol content (TPC), total flavonoid content (TFC), total tannin content (TTC), ferric reducing power (FRAP), 2,2-diphenyl-1-picrylhydrazyl (DPPH) scavenging activity, lipid peroxidation (LOO˙) inhibition, nitric oxide (˙NO) and superoxide radical anion (O_2_˙^−^) scavenging activities of methanol extracts of investigated yacon landraces.

Yacon Landraces	TPC mg GAE/g	TFC mg QE/g	TTC mg TAE/g	FRAP (mg TE/g)	DPPH (IC_50_)	LOO˙ (IC_50_)	˙NO (IC_25_)	O_2_^•−^ (IC_50_)
PER01-M	58.64 ± 1.04	99.59 ± 1.43	8.52 ± 0.67	31.55 ± 0.96	4.21 ± 0.35	0.51 ± 0.05	0.27 ± 0.04	3.81 ± 0.29
PER02-M	84.32 ± 1.57	104.10 ± 2.45	8.97 ± 0.28	45.45 ± 4.91	3.58 ± 0.23	0.61 ± 0.03	0.09 ± 0.01	2.17 ± 0.32
PER03-M	78.64 ± 2.63	78.19 ± 1.59	7.29 ± 0.08	42.53 ± 2.42	4.13 ± 0.50	0.97 ± 0.08	0.11 ± 0.01	3.48 ± 0.25
PER04-M	79.43 ± 1.97	71.93 ± 1.39	6.38 ± 0.03	65.32 ± 6.04	2.08 ± 0.99	0.91 ± 0.06	0.08 ± 0.01	1.85 ± 0.04
PER05-M	85.76 ± 1.65	98.43 ± 3.18	12.94 ± 0.16	45.86 ± 2.24	3.36 ± 0.28	0.94 ± 0.11	0.08 ± 0.01	3.30 ± 0.06
PER06-M	76.56 ± 1.48	82.28 ± 2.22	9.48 ± 0.37	46.16 ± 2.83	3.94 ± 0.45	1.04 ± 0.10	0.14 ± 0.01	2.83 ± 0.20
PER07-M	58.49 ± 0.78	93.00 ± 1.57	7.20 ± 0.48	37.25 ± 4.59	4.39 ± 0.32	0.67 ± 0.03	0.09 ± 0.002	3.40 ± 0.29
PER08-M	60.80 ± 0.69	58.01 ± 0.48	6.45 ± 0.30	39.55 ± 3.46	4.19 ± 0.47	1.26 ± 0.05	0.16 ± 0.02	3.52 ± 0.10
PER09-M	91.07 ± 1.41	91.72 ± 3.46	10.31 ± 0.80	66.80 ± 1.90	2.89 ± 0.12	0.64 ± 0.04	0.05 ± 0.006	1.81 ± 0.08
PER10-M	68.43 ± 3.84	67.59 ± 1.90	14.58 ± 0.47	41.68 ± 4.42	3.37 ± 0.20	0.95 ± 0.08	0.08 ± 0.01	2.11 ± 0.34
PER11-M	85.09 ± 6.73	74.41 ± 3.25	14.28 ± 0.14	53.76 ± 4.66	3.70 ± 0.41	0.52 ± 0.06	0.08 ± 0.02	2.30 ± 0.07
ECU41-M	72.88 ± 3.85	62.43 ± 1.47	9.33 ± 0.55	45.97 ± 0.18	3.55 ± 0.29	1.19 ± 0.13	0.03 ± 0.002	1.40 ± 0.09
ECU43-M	66.78 ± 2.21	57.17 ± 1.21	7.63 ± 0.15	34.83 ± 3.79	3.64 ± 0.25	1.20 ± 0.07	0.09 ± 0.004	1.94 ± 0.05
ECU44-M	75.81 ± 4.87	102.37 ± 5.88	8.13 ± 0.99	49.79 ± 2.80	3.51 ± 0.43	0.83 ± 0.04	0.04 ± 0.001	0.81 ± 0.05

(GAE)/g = mg gallic acid equivalent per gram of dried extract; (QE)/g = mg of quercetin equivalent per gram of dried extract; (TAE)/g = mg of tannic acid equivalent per gram of dried extract; (TE)/g = mg of trolox equivalent per gram of dried extract; IC_50_ and IC_25_ (mg/mL) = 50% and 25% inhibitory concentrations, respectively.

Tannins and flavonoids are secondary metabolites that are widely distributed in the plant kingdom and that exhibit various biological properties [24].

Protein precipitation assays represent the operational property to measure the amount of both condensed and hydrolysable tannins. As reported by Hagerman and Butler [25], bovine serum albumin (BSA) was used as protein in this study. Ferric chloride reacts with phenolic compounds in solution to form complexes with the general formula Fe–(OR)_6_^−3^ where –OR^−^ represents the ionized phenol. The maximum absorption wavelength of the complex is dependent upon the nature of the phenol and the solvent; the complex formed between tannins and ferric chloride in alkaline solution is violet (λ_max_ 510 nm). The triethanolamine, necessary for the maintenance of high pH, was incorporated into the detergent sodium dodecyl suplhate (SDS) solution used to dissolve the tannin-protein complex.

Tannin content was expressed as mg tannic acid equivalent (TAE)/g of dried extract by using a standard curve and results are reported in Table 1.

PER10-M and PER11-M extracts of yacon showed the highest tannin content, 14.58 ± 0.47 and 14.28 ± 0.14 mg TAE/g, respectively. In addition, PER05-M (12.94 ± 0.16 mg TAE/g) and PER09-M (10.31 ± 0.80 mg TAE/g) exhibited a value higher than mean value (9.39 mg TAE/g). The lowest content was observed for PER04-M extract (6.38 ± 0.03 mg TAE/g), followed by PER08-M (6.45 ± 0.30 mg TAE/g). The polyploids landraces, ECU43-M (7.63 ± 0.15 mg TAE/g) and ECU44-M (8.13 ± 0.99 mg TAE/g), showed lower content than their mother plant, ECU41-M (9.33 ± 0.55 mg TAE/g).

The spectrophotometric assay based on aluminum complex formation is one of the most commonly used procedure for the total flavonoid determination.

Aluminum chloride forms acid stable complexes with the C-4 keto group and either the C-3 or C-5 hydroxyl group of flavones and flavonols. In addition, aluminum chloride forms acid labile complexes with the ortho-dihydroxyl groups in the A or B rings of flavonoids [26].

Total flavonoid content was expressed as mg of quercetin equivalent (QE)/g of dried extract (Table 1).

ECU44-M and PER02-M showed the highest content of flavonoids, 104.10 ± 2.45 and 102.37 ± 5.88 mg QE/g, respectively. Flavonoid content, higher than the mean value (841.52 mg QE/g of dried extract) was observed in PER01-M (99.59 ± 1.43 mg QE/g), PER05-M (98.43 ± 3.18 mg QE/g), PER07-M (93.00 ± 1.57 mg QE/g) and PER09-M (91.72 ± 3.46 mg QE/g). The lowest content was observed in the polyploid sample ECU43-M (57.17 ± 1.21 mg QE/g), more similar to its mother plant ECU41-M (62.43 ± 1.47 mg QE/g) and PER08-M (58.01 ± 0.48 mg QE/g).

*In-vitro* assays used in this study are most often colorimetric methods of choice for general determinations of polyphenols, tannins and flavonoids. Although the presence of other constituents in the crude extract could slightly interfere with their quantification, these methods are routinely used [27,28].

### 2.2. In-Vitro Biological Activity

#### 2.2.1. Reducing Power by Ferric Reducing Antioxidant Power (FRAP) Test

Generally, phenolic compounds are responsible for biological activities as antioxidant capacity and inhibition of enzymes involved in common diseases. Antioxidant activity should not be based on a single antioxidant test model and in practice several *in vitro* test procedures are carried out for evaluating antioxidant activities with the samples of interest [29]. The ferric reducing antioxidant power (FRAP) method is based on the reduction of the Fe^3+^ complex of tripyridyltriazine (Fe(TPTZ)^3+^) to the intensely blue colored Fe^2+^ complex (Fe(TPTZ)^2+^) by antioxidants. The method is simple and rapid; it was originally applied to plasma but has been extended to other biological fluids, foods, plant extracts, juices, *etc.*

Results (Table 1) ranged from 31.55 ± 0.96 to 66.80 ± 1.90 mg of Trolox Equivalent (TE)/g of dried extract. Extracts of PER09-M and PER04-M landraces showed the highest reducing power, 66.80 ± 1.90 and 65.32 ± 6.04 mg TE/g of dried extract, respectively. Sample PER01-M (31.55 ± 0.96 mg TE/g) exhibited the lowest value, followed by PER07-M (37.25 ± 4.59 mg TE/g), PER08-M (39.55 ± 3.46 mg TE/g) and ECU43-M (34.83 ± 3.79 mg TE/g). ECU44-M (49.79 ± 2.80 mg TE/g) showed higher reducing power than ECU41-M (45.97 ± 0.15 mg TE/g), its mother plant.

#### 2.2.2. Radical Scavenging Activity by 2,2-Diphenyl-1-picrylhydrazyl (DPPH) Assay

*In vitro* antioxidant tests using free radical traps are relatively straightforward to perform. Among free radical scavenging methods, the one involving 2,2-diphenyl-1-picrylhydrazyl (DPPH) is rapid, simple, highly reproducible and inexpensive in comparison to other test models. The scavenging of the stable DPPH radical is widely used to evaluate the antioxidant activity of phenolic compounds extracted from fruits, vegetables, cereal grains, wine, *etc.* [30]. The method is based on the reduction of methanol DPPH solution in the presence of a hydrogen donating antioxidant, due to the formation of the non-radical form DPPH-H. The extracts were able to reduce the stable radical DPPH to the yellow colored diphenylpicrylhydrazine, in a concentration-dependent manner. Results were expressed as IC_50_ (Table 1) and all extracts showed a fair DPPH scavenging activity, lower than Trolox (used as standard) with an IC_50_ of 0.08 ± 0.002 mg/mL. The most active of the yacon landraces was PER04-M (IC_50_ = 2.08 ± 0.99 mg/mL) followed by PER09-M (IC_50_ = 2.89 ± 0.12 mg/mL). PER01-M (IC_50_ = 4.21 ± 0.35 mg/mL), PER08-M (IC_50_ = 4.19 ± 0.47 mg/mL) and PER07-M (IC_50_ = 4.39 ± 0.32 mg/mL) showed the lowest activity. Methanol extract of polyploids samples (ECU43-M and ECU44-M) and their mother plant (ECU41-M) displayed similar DPPH-scavenging activity.

#### 2.2.3. Nitric Oxide (˙NO) Radical Scavenging Activity

˙NO is known to be a ubiquitous free radical, being distributed in tissues or organ systems and supposed to have a vital role in neuromodulation or as a neurotransmitter in the central nervous system CNS. In addition to reactive oxygen species, nitric oxide is also implicated in inflammation, cancer and other pathological conditions. In the *in vitro* assay performed, sodium nitroprusside is known to decompose in aqueous solution at physiological pH (7.2) producing ˙NO [31]. Under aerobic conditions, ˙NO reacts with oxygen to produce stable products (nitrate and nitrite), the quantities of which can be determined using Griess reagent [32]. The Griess reaction is based on the two-step diazotization reaction in which acidified NO_2_^−^ produces a nitrosating agent, which reacts with sulfanilic acid to produce the diazonium ion. This ion is then coupled to *N*-(1-naphthyl) ethylenediamine to form a chromophoric azo-derivative, absorbing at 560 nm [33].

The ability to inhibit nitric oxide production was concentration-dependent and, as far as we know, not reported yet for yacon leaf extract. The IC_50_ was not reached with the tested concentrations, so the results were expressed as IC_25_ (mg/mL) and are reported in Table 1. Ascorbic acid was used as reference, showing a IC_25_ value of 0.06 ± 0.003 mg/mL. All extracts demonstrated to possess nitric oxide scavenging activity. Foliar extracts of ECU41-M (IC_25_ = 0.03 ± 0.002 mg/mL) and ECU44-M (IC_25_ = 0.04 ± 0.001 mg/mL) showed the highest scavenging activity; also PER09-M (IC_25_ = 0.05 ± 0.006 mg/mL) reported a good scavenging activity, higher than ascorbic acid. Sample PER01-M, with the highest IC_25_ value (0.27 ± 0.04 mg/mL), was the less active.

#### 2.2.4. Superoxide Radical (O_2_˙^−^) Scavenging Activity

The production of reactive oxygen species, such as superoxide (O_2_˙^−^), by mitochondria is a major cause of cellular oxidative stress and it contributes to many pathological conditions [34]. Superoxide anion radical (O_2_˙^−^) is generated in aerobic cells due to electron leakage from the electron transport chain.

In this study, *in vitro* superoxide anions are generated by phenazine methosulfate-β-nicotinamide adenine dinucleotide (PMS-NADH) system. The superoxide scavenging capacity of methanol extracts was quantified by their ability to inhibit nitrotetrazolium blue chloride (NBT) reduction by superoxide.

Scavenging activity was, also in this case, concentration-dependent and the activity of ascorbic acid, used as reference, was compared with the samples by IC_50_ values. Ascorbic acid displayed the strongest activity, showing the lowest IC_50_ (0.28 ± 0.04 mg/mL). Methanol extract of ECU44-M exhibited the lowest IC_50_ value among investigated landraces (IC_50_ = 0.81 ± 0.05 mg/mL), so it was the most active against superoxide radical obtaining a comparable value to ascorbic acid, used as positive control. Also PER09-M (IC_50_ = 1.81 ± 0.08 mg/mL), PER04-M (IC_50_ = 1.85 ± 0.04 mg/mL) and PER02-M (IC_50_ = 2.17 ± 0.32 mg/mL) showed a good superoxide anion scavenging activity, with an IC_50_ lower than mean value (IC_50_ = 2.48 mg/mL). PER01-M was the weakest physiological radicals scavenger, both against superoxide radical (IC_50_ = 3.81 ± 0.29 mg/mL) and ˙NO.

#### 2.2.5. Lipid Peroxidation (LOO˙) Inhibition Test

Lipid peroxidation is thought to be an important factor in the pathophysiology of a number of diseases. Lipid peroxidation is probably the most extensively investigated free radical-induced process [35]. In this study, lipid peroxidation inhibition was assessed by evaluating the formation of conjugated dienes, structures with a double-single-double bond (–C=C–C=C–) arrangement, absorbing UV light in the wavelength range 230–235 nm [36,37,38].

This test was used in the antioxidant activity evaluation of the methanol extracts of yacon. Results were expressed as IC_50_ (Table 1). Butylated hydroxytoluene (BHT) was used as reference, showing the highest inhibition of lipid peroxidation (IC_50_ = 0.01 ± 0.001 mg/mL). All extracts displayed IC_50_ values higher than the reference compound. Among all extracts, the most able to scavenge LOO˙ were PER01-M (IC_50_ = 0.51 ± 0.05 mg/mL) and PER11-M (IC_50_ = 0.52 ± 0.06 mg/mL); similar values were also observed with PER02-M (IC_50_ = 0.61 ± 0.03 mg/mL), PER09-M (IC_50_ = 0.64 ± 0.04 mg/mL) and PER07-M (IC_50_ = 0.67 ± 0.03 mg/mL), whereas the lowest capacity was found for PER08-M (IC_50_ = 1.26 ± 0.05 mg/mL), ECU43-M (IC_50_ = 1.20 ± 0.07 mg/mL) and ECU41-M (IC_50_ = 1.19 ± 0.13 mg/mL).

#### 2.2.6. Inhibition Activity against α-Glucosidase and α-Amylase Enzymes

Medicinal plants and herbal extracts containing glycosides, flavonoids, tannins, *etc.*, have been reported to demonstrate antidiabetic [39] and anticholinesterase activities [40]. One therapeutic approach for treating diabetes is to decrease post-prandial hyperglycemia. This is done by hindering the absorption of glucose through inhibition of the carbohydrate hydrolyzing enzymes, α-amylase and α-glucosidase, in the digestive tract. In our investigation, the inhibitory activity of crude methanol extracts against both mentioned enzymes was carried out. Previous study reported only the α-glucosidase inhibitory activity of some isolated constituents from yacon leaves [6].

**Figure 1:**
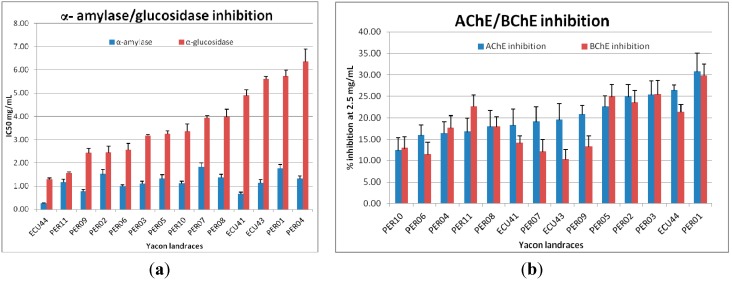
(**a**) α-Amylase and α-glucosidase inhibition activity and (**b**) acetylcholinesterase (AChE) and butyrylcholinesterase (BChE) inhibition of tested yacon extracts.

Results were expressed as IC_50_. Acarbose was used as reference drug showing IC_50_ values lower than 0.20 mg/mL in both inhibition assays. Data obtained showed that methanol extracts of yacon leaves were stronger inhibitors of α-amylase than α-glucosidase, as presented in Figure 1a. Among yacon extracts, the most active was found to be ECU44-M landrace for both enzyme inhibitory activities (IC_50_ = 0.26 ± 0.02 mg/mL for α-amylase inhibition and 1.30 ± 0.04 mg/mL for α-glucosidase); ECU41-M and PER09-M extracts act as α-amylase inhibitors; additionally, PER11-M can be considered a moderate inhibitor of α-glucosidase; on the other hand, the less active against both enzymes were PER01-M and PER04-M extracts.

According to previous study [6], our investigation reported that methanol extracts moderately inhibited α-amylase and α-glucosidase and this result can, at least partially, explain the traditional use of yacon leaves for treating hyperglycemia.

#### 2.2.7. Inhibition Activity against Acetylcholinesterase and Butyrylcholinesterase Enzymes

For the first time yacon extracts were tested to evaluate their capacity to inhibit the cholinesterase enzymes. The crucial role of the cholinesterases in neural transmission makes them a primary target of a large number of cholinesterase-inhibiting drugs and this has relevance to the treatment of neurodegenerative disorders.

The method for the screening of ChE inhibitory activity from natural resources based on Ellman’s reactions has been reported [41]. In 1959, Ellman introduced 5,5′-dithio-bis-(2-nitrobenzoic acid), also known as DTNB, as a versatile water-soluble compound for quantitating free sulfhydryl groups in solution. A solution of this compound produces a measurable yellow-colored product when it reacts with sulfhydryl. Consequently, Ellman’s reagent is very useful as a sulfhydryl assay reagent because of its specificity for –SH groups at neutral pH, high molar extinction coefficient and short reaction time. DTNB reacts with a free sulfhydryl group to yield a mixed disulfide and 2-nitro-5-thiobenzoic acid (TNB). The target of DTNB in this reaction is the conjugated base (R–S–) of a free sulfhydryl group.

In the *in vitro* assays, AChE and BChE efficiently catalyze the hydrolysis of acetyl- and butyryl-thiocholine (AcSCh and BuSCh), sulfur analogs of their respective natural substrate, acetylcholine. Upon hydrolysis, these substrate analogs produce acetate (or butyrate) and thiocholine. Thiocholine in the presence of the highly reactive DTNB ion reacts to generate the yellow 5-thio-2-nitrobenzoate anion. The yellow color can be quantified at 405 nm [41].

Galantamine, a natural cholinesterase inhibitor used in therapeutics, was used as reference and its capacity to inhibit the two enzymes involved in Alzheimer’ disease was measured: 93.5% ± 1.2% AChE inhibition and 68.3% ± 3.4% BChE inhibition, at only 0.01 mg/mL.

Yacon landraces demonstrated a concentration-dependent inhibition, but unfortunately the weak activity of the extracts did not allow to reach, and consequently calculate, the IC_50_. In fact, results were expressed as % of inhibition at 2.5 mg/mL, and they showed an inhibition lower than 35% against both enzymes.

Among them, PER01-M showed the strongest capacity against both AChE and BChE, in this case similar values have been found: 30.87% ± 4.20% and 29.83% ± 2.68%, respectively. Most of the yacon landraces displayed the same effect against both cholinesterases, showing an inhibition lower than 25% at the tested concentration.

### 2.3. High Perfomance Liquid Chromatography-Diode Array Detector Analysis

Phenolic compounds have characteristic UV–Vis spectral properties that are unique for each of the different classes of phenolics, and may therefore be used for tentative identification of the class of a phenolic compound.

Phytochemical studies of yacon leaves showed the presence of several melampolide-type sesquiterpene lactones such as sonchifolin, uvedalin, enhydrin, fluctuanin [42] and phenolic compounds, mainly chlorogenic acid and other caffeic derivatives, have been identified in different extracts of yacon leaves together with some flavonoids [7,12,43,44,45].

High perfomance liquid chromatography-diode array detector (HPLC-DAD) analysis of methanol extract of yacon leaves confirmed the presence of rutin and caffeic acid. Other compounds were identified by comparing with the results obtained with *Cynara cardunculus* extract analyzed under the same conditions [46]. Methanol extract of *C. cardunculus* is characterized to be rich in caffeoylquinic acids and analytical parameters allowed to identify chlorogenic acid, 3,5-*O*-dicaffeoylquinic acid, 1,5-*O*-dicaffeoylquinic acid and 4,5-*O*-di-caffeoylquinic acid. The compound 1,5-*O*-dicaffeoylquinic acid was identified for the first time in yacon leaves.

**Figure 2:**
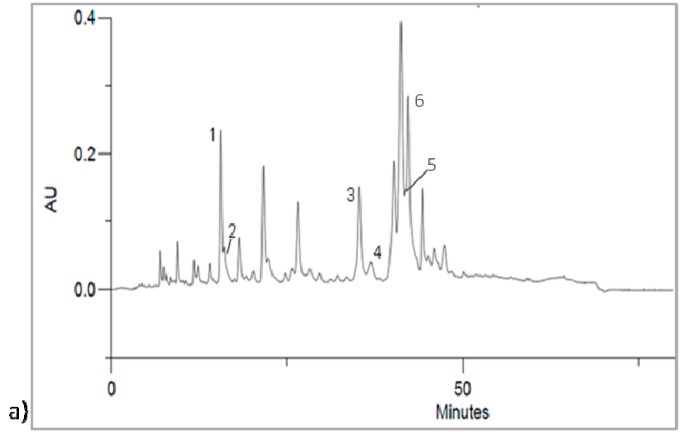

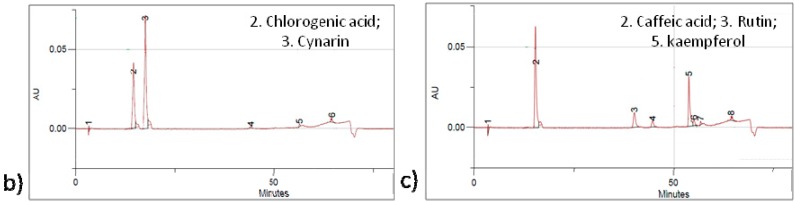
(**a**) Chromatogram obtained by high perfomance liquid chromatography-diode array detector (320 nm) of methanol extract of PER02 landrace. **1**: Chlorogenic acid (Rt = 14.95 min), **2**: Caffeic acid (Rt = 15.18 min), **3**: 3,5-di-*O*-caffeoylquinic acid (Rt = 33.86 min), **4**: 1,5-di-*O*-caffeoylquinic acid (Rt = 35.60 min), **5**: Rutin (Rt = 39.68 min), **6**: 4,5-di-*O*-caffeoylquinic acid (Rt = 41.29 min); (**b**) HPLC chromatogram (320 nm) of the standards: chlorogenic acid (Rt = 14.72 min) and cynarin (Rt = 17.62 min); (**c**) HPLC chromatogram (320 nm) of the standards caffeic acid (Rt = 15.31 min), rutin (Rt = 39.59 min) and kaempferol (Rt = 53.38 min).

Analyzing HPLC chromatograms, it is possible to observe a similar phytochemical profile; it was just reported a representative chromatogram (Figure 2). Quantitative analysis demonstrated that all samples contained a high amount of phenolic acids. Results are reported in Table 2.

Chlorogenic acid is the most abundant compound and it was already found both in leaves and root of yacon [42,47]. It ranged from 13,033.96 ± 121.60 mg of chlorogenic acid/kg of extract (PER09-M) to 1383.44 ± 2.16 mg of chlorogenic acid/kg of extract (PER01-M). High content of this compound was found in PER11-M (10,069.14 ± 64.17 mg of chlorogenic acid/kg of extract) and PER10-M (9930.52 ± 58.32 of chlorogenic acid/kg of extract). Among identified caffeoylquinic acids, high content of 3,5- and 4,5-*O*-dicaffeoylquinic acids was also observed, with mean values of 4018.70 and 2424.57 mg cynarin/kg, while 1,5-*O*-dicaffeoylquinic acid was found in small amounts in the extracts (Table 2).

Among all methanol extracts, PER09-M contained the highest amount of secondary metabolites, both flavonoids and phenolic acids, and in particular, it was found to be rich in caffeic and chlorogenic acids. The ECU44 landraces showed large amounts of phenylpropanoid derivatives and, among all identified compounds, caffeoylquinic acids are abundant. Sample ECU41-M, together with its polyploids, ECU43-M and ECU44-M, demonstrated the highest content of 3,5-*O*-dicaffeoylquinic acid. PER01-M was the sample with the lowest content of secondary metabolites, in particular it showed a low content of phenylpropanoid acids, but together with PER02-M, contained a high amount of rutin and flavonoid derivatives. PER08-M showed the lowest content of rutin and flavonoids, according to the results obtained by colorimetric assay.

**Table 2:** HPLC quantification of identified compounds in yacon landraces.

Yacon Landraces	Chlorogenic Acid (mg Chlorogenic Acid/kg)	Caffeic Acid (mg Caffeic Acid/kg)	3,5-*O-*di-Caffeoylquinic Acid (mg Cynarin/kg)	1,5-*O*-di-Caffeoylquinic Acid (mg Cynarin/kg)	4,5-*O*-di-Caffeoylquinic Acid (mg Cynarin/kg)	Rutin (mg Rutin/kg)
PER01-M	1383.44 ± 2.16	319.73 ± 2.90	606.47 ± 5.97	222.24 ± 5.28	937.93 ± 2.84	951.06 ± 3.51
PER02-M	3010.99 ± 65.66	452.39 ± 5.05	1741.36 ± 42.92	442.99 ± 1.02	1965.34 ± 31.88	1193.60 ± 36.31
PER03-M	3180.23 ± 23.86	476.02 ± 8.78	2555.21 ± 35.03	303.06 ± 11.86	1766.98 ± 169.29	608.75 ± 8.57
PER04-M	5580.74 ± 26.86	635.86 ± 16.06	4069.10 ± 58.98	673.27 ± 23.67	2857.90 ± 139.97	548.03 ± 10.98
PER05-M	5004.72 ± 18.31	697.12 ± 14.54	3345.68 ± 36.06	425.54 ± 25.32	1915.80 ± 28.12	668.23 ± 5.81
PER06-M	3727.59 ± 35.54	802.68 ± 24.60	2587.01 ± 24.11	597.77 ± 10.78	1807.18 ± 22.20	476.23 ± 5.73
PER07-M	5965.41 ± 45.99	622.39 ± 28.61	1166.08 ± 31.01	448.72 ± 24.00	2868.39 ± 33.65	657.37 ± 13.51
PER08-M	6799.45 ± 128.33	732.93 ± 21.12	3061.57 ± 46.59	623.67 ± 66.17	1984.75 ± 35.12	387.17 ± 24.63
PER09-M	13,033.96 ± 121.60	1133.52 ± 36.37	6339.79 ± 49.55	663.85 ± 80.83	3998.25 ± 37.18	803.94 ± 57.00
PER10-M	9930.52 ± 58.32	1060.62 ± 28.83	4892.73 ± 24.78	437.50 ± 28.59	1880.87 ± 28.27	958.23 ± 48.19
PER11-M	10,069.14 ± 64.17	920.17 ± 14.16	4792.38 ± 58.39	534.08 ± 63.73	2671.80 ± 42.16	870.33 ± 21.55
ECU41-M	8012.71 ± 52.60	665.86 ± 16.21	5479.88 ± 61.82	255.33 ± 27.66	2378.10 ± 22.26	1347.27 ± 50.41
ECU43-M	8523.77 ± 61.15	863.10 ± 21.50	5934.75 ± 61.58	367.16 ± 36.69	3094.95 ± 34.10	974.84 ± 28.70
ECU44-M	8009.38 ± 48.36	1047.42 ± 34.25	9689.76 ± 101.26	677.11 ± 10.01	5218.88 ± 47.30	992.15 ± 63.60

### 2.4. Statistical Analysis

To compare data obtained by different chemical method used to evaluate extract antioxidant activity, a new concept, relative antioxidant capacity index (RACI) [48], was proposed. This concept provides a more comprehensive comparison when several samples are analyzed.

All methods used for antioxidant activity determination together with TPC were included in RACI calculation. Recently, TPC assay has been proposed for the measurement of total reducing capacity of samples [49].

**Figure 3:**
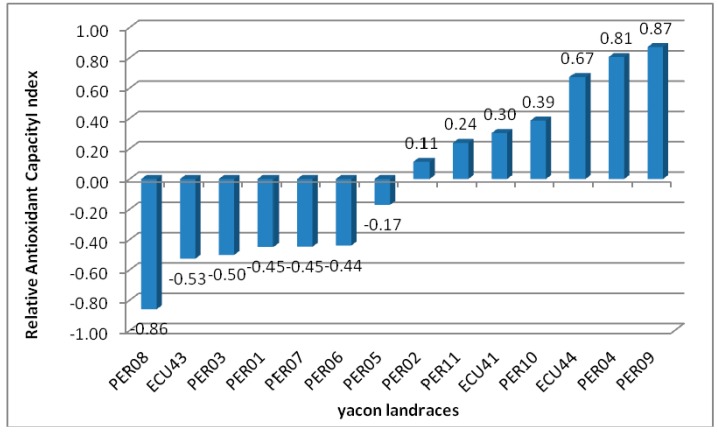
Relative antioxidant capacity index of investigated yacon landraces.

Results of antioxidant activity expressed as IC_25_ or IC_50_ were converted in 1/IC_25_ and 1/IC_50_, before the RACI calculation. Data of relative antioxidant activity were represented as histograms (Figure 3). According to obtained results, methanol extract of PER09 sample showed the highest index, 0.87, followed by PER04-M (0.81). Positive value was displayed by ECU44-M (0.67), PER10 (0.39), ECU41 (0.30), PER11 (0.24) and PER02 (0.11). The yacon landrace extract showing the lowest RACI was PER08-M (−0.86). The extracts with the highest RACI contribute in significant manner to the complex antioxidant activity, although they had a different attitude depending on the various used methods.

To understand the correlation among all studied variables, total polyphenols, flavonoid and tannin content and biological activity (reducing power, radical-scavenging activity, lipid peroxidation, inhibition of AChE, BChE, α-glucosidase and α-amylase), Pearson’s correlation coefficient was calculated. The analysis was conducted using averaged values of each variable and results are reported in Table 3. Data expressed as IC_50_ and IC_25_, show a negative correlation. The highest correlation was observed between total polyphenol content and reducing power (r = 0.77). Polyphenols seem to be mostly involved in scavenging activity against DPPH (r = −0.58), superoxide radical (r = −0.39) and nitric oxide (r = −0.53) demonstrating the ability of these compounds to quench radicals and reduce the oxidative and nitrosative stress. Flavonoids were the main contributors to lipid peroxidation inhibition (r = −0.72). Pearson correlation between phenolic content and enzymatic inhibition was also calculated. Polyphenols showed to be moderately related to α-glucosidase (r = −0.51) and α-amylase (r = −0.41) enzyme inhibitions, as well flavonoids can contribute to AChE and BChE inhibition (r = 0.66 and r = 0.51, respectively), as previously reported [50]. Among all investigated activities, tannin content demonstrated the highest correlation with α-glucosidase inhibition (r = −0.45).

**Table 3:** Pearson correlation among total polyphenol content (TPC), total tannin content (TTC), total flavonoid content (TFC) and antioxidant activity (reducing power, 2,2-diphenyl-1-picryl hydrazyl (DPPH), ˙NO, O_2_˙^−^ scavenging activity and lipid peroxidation inhibition) and enzymatic inhibition (AChE, BChE, α-amylase and α-glucosidase enzymes).

Variables	TPC	TTC	TFC
Reducing power (FRAP)	0.77	0.14	0.08
DPPH scavenging activity	−0.58	−0.12	0.06
Lipid peroxidation inhibition (LOO˙)	−0.24	−0.27	−0.72
˙NO scavenging activity	−0.53	−0.23	0.11
O_2_˙^−^ scavenging activity	−0.39	−0.11	0.09
AChE inhibition	−0.02	−0.32	0.66
BChE inhibition	0.15	0.05	0.51
α-Amylase inhibition	−0.41	−0.13	0.12
α-Glucosidase inhibition	−0.51	−0.45	−0.41

**Figure 4:**
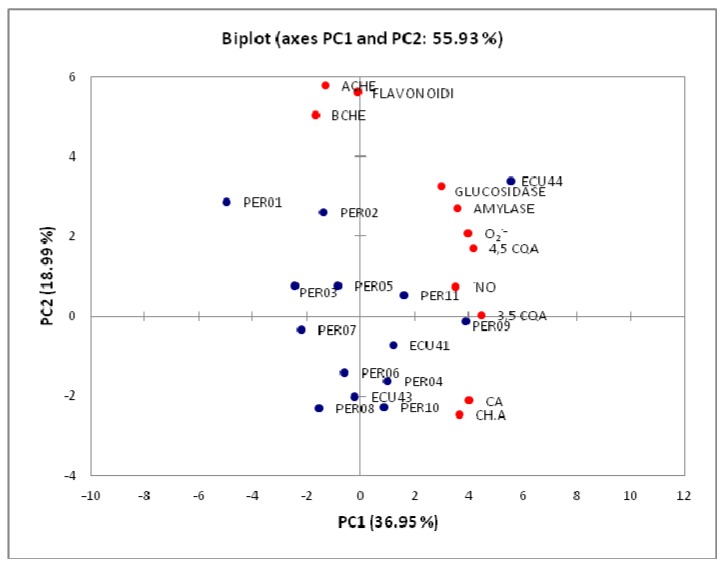
Principal component analysis (PCA) among biological activity and chemical profile of investigated yacon landraces.

In order to discriminate the yacon landraces on the basis of the chemical profile and biological activity, data were normalized and principal component analysis (PCA) was conducted on the correlation matrix. In this study, PCA was used for a better visualization of data sets obtained from the determinations of all studied variables as previously reported [51,52]: content of total phenols, flavonoids, tannin, chlorogenic acid (CHA), caffeic acid (CA), rutin, 1,5-*O-*di-caffeoylquinic acid (1,5-CQA), 4,5-*O-*di-caffeoylquinic acid (4,5-CQA), 3,5-*O-*di-caffeoylquinic acid (3,5-CQA), as well as biological activity (reducing power, radical-scavenging activity, lipid peroxidation, inhibition of AChE, BChE, α-glucosidase and α-amylase). The use of this unsupervised classification method often permits a simple representation of yacon landraces data and their correlations. The first two principal components (PCs) described 55.93% of the initial data variability (PC1 36.95% and PC2 18.99%) (Figure 4). Further components explained 13.24%, 10.08% and 7.43% of variance. The loading of PC1 had a strong positive correlation with nitric oxide and superoxide (SO) scavenging activity, α-amylase and α-glucosidase inhibitions and with the content of CHA, CA, 3,5-CQA and 4,5-CQA. The strong positive loadings of PC2 were total flavonoid content and inhibition of cholinesterase enzymes. Compounds 4,5-CQA and 3,5-CQA showed good correlation with nitric oxide and superoxide scavenging activity and α-amylase and α-glucosidase inhibition. These compounds were found in high amount in ECU44-M and results were confirmed by PCA analysis. As reported by PCA analysis, flavonoid content, abundant in ECU44-M, PER01-M and PER02-M, are involved in the inhibition of cholinesterase enzymes.

## 3. Experimental Section

### 3.1. Chemicals

Chloroform, *n*-hexane, methanol, hydrochloric acid and glacial acetic acid were purchased from Carlo Erba (Milano, Italy). Ethanol, potassium di-hydrogen phosphate, di-sodium tetraborate, iron (II) sulfate (FeSO_4_·7H_2_O) and *N*-(1-naphthyl) ethylenediamine dihydrochloride were obtained from Merck (Darmstadt, Germany). Folin–Ciocalteu reagent 2N, sodium carbonate, sodium acetate trihydrate, 2,4,6-tripyridyl-*s*-triazine (TPTZ), iron (III) chloride (FeCl_3_·6H_2_O), 2,2-diphenyl-1-picryl hydrazyl (DPPH) radical, and standards as 6-hydroxy-2,5,7,8-tetramethylchroman-2-carboxylic acid (Trolox) and gallic acid were purchased from Sigma–Aldrich (Milan, Italy). Butylated hydroxytoluene (BHT, 2,6-bis(1,1-dimethylethyl)-4-methylphenol), β-nicotinamide adenine dinucleotide reduced form (NADH), phenazine methosulfate (PMS), nitrotetrazolium blue chloride (NBT), 5,5′-dithio-bis(2-nitrobenzoic acid) (DTNB), sodium nitroprusside dehydrate (SNP), linoleic acid, l-ascorbic acid and sulphanilamide, acetylcholinesterase (AChE) from electric eel (type VI-s, lyophilized powder), acetylthiocholine iodide (ATCI), butyrylcholinesterase (BChE) from equine serum (lyophilized powder), *S*-butyrylthiocholine chloride (BTCC), α-glucosidase (type I from baker’s yeast) and 4-nitrophenyl α-d-glucopyranoside (PNP-G) were purchased from Sigma (St. Louis, MO, USA). Trizma hydrochloride (Tris-HCl) and bovine serum albumin (BSA) were provided from Sigma–Aldrich (Steinheim, Germany).

Chlorogenic acid, caffeic acid, cynarin, kaempferol and rutin used as standards were purchased from Extrasynthese (Genay, France). HPLC-grade methanol and formic acid were acquired from Merck (Darmstadt, Germany). Water was deionized using a Milli-Q water purification system (Millipore, Bedford, MA, USA).

### 3.2. Plant Material

In this study, dried foliar tissues from fourteen landraces of *Smallanthus sonchifolius* (named PER01–PER11, ECU41, ECU43 and ECU44, as assigned in a previous study [53]) were analyzed to investigate the phenolic content and biological properties. A voucher specimen for each landrace (numbered CZU-ECU41, ECU43, ECU44 and PER*n*-11, where *n* is the number of the landrace as named above) is deposited in herbarium of the Faculty of Tropical Agriculture, Czech University of Agriculture, Prague. The plant material has been acquired since 1993 from different parts of the world and selected for their morphological traits [54]. They are maintained and cultivated under the field conditions of trial plots at the Faculty of Tropical AgriSciences (FTA, former Institute of Tropics and Subtropics), Czech University of Life Sciences Prague (CULS) [53].

### 3.3. Preparation of Plant Extracts

Dried leaves of all yacon landraces were coarsely powered by mortar and pestle and extracted by maceration technique with frequent agitation for three times with the same solvent (ratio 1:8 *w*/*v*). Solvents with increasing polarity, *n*-hexane, chloroform (CHCl_3_), mixture CHCl_3_:methanol (MeOH) in ratio 9:1 and MeOH were used. The mixtures were filtered by cellulose filter paper (17–25 μm) and the combined liquids were evaporated to dryness under reduced pressure by using rotary evaporator. The extracts were kept in the dark at room temperature until use. The percentage of yield extracts was calculated as % yield = (Weight of dried extract/Initial weight of dried foliar tissue) × 100.

### 3.4. Total Polyphenol, Tannin and Flavonoid Content

#### 3.4.1. Total Polyphenol Content

The total phenolic (TPC) content was determined by Folin–Ciocalteu reagent [55]. Briefly, 75 μL of diluted extract and 425 μL of distilled water was added to 500 μL Folin–Ciocalteu reagent and 500 μL of Na_2_CO_3_ (10% *w*/*v*). The mixture was mixed and incubated for 1 h in the dark at room temperature. After incubation, the absorbance was measured at 723 nm using a UV–Vis spectrophotometer (DU 640 Spectrophotometer, Beckman, Brea, CA, USA). The total phenolic content was expressed as mg gallic acid equivalent (GAE)/gof extract.

#### 3.4.2. Total Tannin Content

To 250 μL of different concentrations of extract (10 mg/mL), 500 μL of bovine serum albumin solution in 0.2 mol/L acetic buffer, pH 5.0 with 0.17 mol/L NaCl (1 mg/mL) was added and mixed carefully [25]. After 15 min, the samples were centrifuged at 5000 g for 15 min. The supernatant was removed, and the pellet dissolved in 1 mL of aqueous solution containing 1% SDS and 4% triethanolamine. Then, 250 μL of 0.01 mol/L FeCl_3_ in 0.01 mol/L HCl was added. After 30 min the absorbance was recorded at 510 nm. Results were expressed as mg of tannic acid equivalent/g of extract (mg TAE/g of extract).

#### 3.4.3. Total Flavonid Content

An aliquot (150 μL) of extract at 10.00 mg/mL was added to 45 μL of 5% NaNO_3_ into microcentrifuge tube. After 5 min, 90 μL of 1% AlCl_3_ was added and at the 6th minute, 300 μL of 1 M NaOH solution was added and the total volume was made up to 1.5 mL with distilled water. The solution was mixed well and the absorbance was measured against reagent blank at 510 nm after 10 min of incubation at room temperature [56]. Quercetin was used as standard to plot the calibration curve. The total flavonoid content was expressed as mg of quercetin equivalent/g of extract (mg QE/g of extract).

### 3.5. Biological Activity

#### 3.5.1. Reducing Power

Reducing power of the extracts was determined by ferric reducing ability power test (FRAP) [57]. The stock solution included 300 mM acetate buffer, pH 3.6, 10 mM TPTZ (2,4,6-tripyridyl-*s*-triazine) solution in 40 mM HCl, and 20 mM FeCl_3_·6H_2_O solution. The working solution was prepared by mixing acetate buffer, TPTZ and FeCl_3_·6H_2_O (10:1:1). Plant extracts (150 μL) were allowed to react with 2850 μL of the FRAP solution for 40 min at 37 °C. Readings of the colored product (ferrous tripyridyltriazine complex) were taken at 593 nm. Trolox was used as standard and results were expressed in mg of trolox equivalent (TE)/g of dried sample.

#### 3.5.2. DPPH Scavenging Activity

Radical-scavenging activity was evaluated by using DPPH test [58]. Briefly, 80 μL of plant extracts were added to 1420 μL of DPPH (2,2-diphenyl-1-picrylhydrazyl) solution in a microcentrifuge tubes and left in the dark. The absorbance was monitored every 30 min until 90 min at 515 nm by using spectrophotometer (UV 640 Spectrophotometer). Results were expressed as IC_50_ and compared with the Trolox, used as standard.

#### 3.5.3. Nitric Oxide (˙NO) Radical Scavenging Activity

The capacity to scavenge ˙NO was evaluated spectrophotometrically in a Multiskan Ascent plate reader (Thermo Electron Corporation, Shanghai, China), according to a previously described procedure [33], with different concentration of extracts. ˙NO was generated *in vitro* from sodium nitroprussiate dehydrate (SNP) and measured by the Griess reaction. SNP solution (6 mg/mL) was prepared in phosphate buffer (KH_2_PO_4_ 100 mM, pH 7.4) and mixed with the same volume (100 μL) of different concentrations of extracts, in a 96-wells plate. The mixture was further incubated at room temperature for 1 h under light. After that, 100 μL of Griess reagent (1:1 mixture (*v*/*v*) of 1% sulfanilamide and 0.1% *N*-(1-naphthyl) ethylenediamine in 2% H_3_PO_4_) was added and the mixture was further incubated for 10 min in the dark. The absorbance was read at 560 nm. Results were expressed as IC_50_ and ascorbic acid was used as positive control.

#### 3.5.4. Superoxide Radical (O_2_˙^−^) Scavenging Activity

The effect of the extract on the superoxide radical-induced reduction of NBT was monitored spectrophotometrically in a Multiskan Ascent plate reader (Thermo Electron Corporation), in kinetic function, at 560 nm. Superoxide radicals were generated by the phenazine methosulfate-β-nicotinamide adenine dinucleotide (PMS-NADH) system, as previously reported [33]. Several dilutions of sample (50 μL) or phosphate buffer as control, NADH (50 μL) and NBT (150 μL) were put in the 96-well plate. The reaction was started by adding PMS (50 μL) to the mixture. The assay was conducted at room temperature at 560 nm for 2 min. For each extract, five different concentrations were tested. Results were expressed as IC_50_ and ascorbic acid was used as positive control.

#### 3.5.5. Lipid Peroxidation Inhibition (LOO˙ Radical)

Lipid peroxyl radical (LOO˙) was generated as proposed by Ferreres *et al.* [59] with slight modifications. The reaction mixture contained 250 μL of 5 mM linoleic acid, 150 μL of Tris-HCl (100 mM, pH 7.5), 50 μL of 4 mM FeSO_4_·7H_2_O and 50 μL of serial dilutions of extracts prepared in distilled H_2_O. Linoleic acid peroxidation was initiated by the addition of 50 μL of 5 mM ascorbic acid, and the mixture was immediately incubated for 1 h at 37 °C. After the incubation period, 3 mL of ethanol was added to each test tube. The mixtures were vortexed and the absorbance was immediately measured at 233 nm in a Helios α (Unicam) spectrophotometer, at room temperature. Results were expressed as IC_50_ and BHT was used as positive control.

#### 3.5.6. α-Amylase and α-Glucosidase Inhibitory Activity

Different concentration of each sample extract (100 μL) and 100 μL of 0.02 M sodium phosphate buffer (pH 6.9 with 0.006 M NaCl) containing α-amylase solution (0.5 mg/mL) were incubated at 25 °C for 10 min. After pre-incubation, 100 μL of a 1% starch solution in sodium phosphate buffer was added to each tube at timed intervals. The reaction mixtures were then incubated at 25 °C for 10 min. The reaction was stopped with 200 μL of dinitrosalicylic acid color reagent. The test tubes were then incubated in a boiling water bath for 5 min and cooled to room temperature. The reaction mixture was then diluted after adding 3 mL of distilled water, and absorbance was measured at 540 nm. The absorbance of blanks (enzyme solution was added during the boiling) and a control (buffer in place of sample extract) were recorded [60]. Analyses were performed in triplicate and the final extract absorbance (540 nm) was obtained by subtracting its corresponding sample blank reading.

The effect on α-glucosidase was assessed in 96-well plates, using a procedure previously reported [61]. Briefly, each well contained 2.5 mM PNP-G (100 μL) in phosphate buffer pH 7.0 (150 μL) and methanol extract at different concentrations (50 μL). The reaction was initiated by the addition of 0.28 U/mL enzyme (20 μL) and the plates were incubated at 37 °C for 10 min. The absorbance at 400 nm was measured in a Multiskan Ascent plate reader (Thermo Electron Corporation). Results were expressed as IC_50_ calculated from three independent tests, performed in triplicate and acarbose was used as positive control in both assays.

#### 3.5.7. Acetylcholinesterase (AChE) and Butyrylcholinesterase (BChE) Inhibitory Activity

The inhibition of AChE activity was determined based on Ellman’s method, as previously reported [61]. In this assay, 25 μL of acetylthiocholine iodide (15 mM), 125 μL of DTNB (3 mM), 25 μL of buffer B (50mM Tris-HCl, pH 8 containing 0.1% BSA) and 50 μL of each test extract solution at the different concentrations were mixed. The mixture was monitored at 405 nm. The reaction was started by adding 25 μL of 0.44 U/mL AChE. The absorbance was measured at 405 nm and the rates of reactions were calculated by Ascent Software version 2.6 (Thermo Labsystems Oy, Vantaa, Finland). The BChE inhibition assay was performed in a similar way [62] using 25 μL of 15 mM butyrylthiocholine chloride as substrate and 0.1 U/mL of BChE as enzyme. Three independent assays were performed in triplicate at different concentrations.

### 3.6. HPLC-DAD Qualitative and Quantitative Analyses

Redissolved methanol extract (50 mg/mL) was analyzed on an analytical HPLC-DAD unit (Gilson) using a Luna C18 column (250 × 4.6 mm, 5 μm particle size; Phenomenex, Macclesfield, UK). The mobile phase consisted of two solvents: water-formic acid (5%) (A) and methanol (B), starting with 5% B and using a gradient to obtain 15% B at 3 min, 25% B at 13 min, 30% B at 25 min, 35% B at 35 min, 45% B at 42 min, 55% B at 47 min, 75% B at 56 min and 100% B at 60 min. The flow rate was 0.9 mL/min. Spectral data from all peaks were collected in the range of 200–400 nm, and chromatograms were recorded at 320 nm for hydroxycinnamic acids and at 350 nm for flavonoids. The data were processed on Unipoint System software (Gilson Medical Electronics, Villiers le Bel, France).

Whenever available, reference standards of phenolics were used to substantiate the identification of the peaks. Phenolic compounds, which were not available as standard reference materials were tentatively identified according to the literature [62] and confirmed by matching their retention time and UV spectra using the same chromatographic condition of a previous work conducted by our team [63].

Phenolic compounds quantification was achieved by the absorbance recorded in the chromatograms relative to external calibration standards.

Phenolic acid derivatives (320 nm) were quantified as caffeic acid, 1,5-di-*O*-caffeoylquinic, 3,5-di-*O*-dicaffeoylquinic acid and 4,5-di-*O*-caffeoylquinic acid were quantified as cynarin (1,3-*O*-dicaffeoylquinic acid, 320 nm), flavonoid derivatives were quantified as kaempferol (350 nm). The other compounds were quantified as themselves.

### 3.7. Statistical Analysis

A minimum of three independent experiments were carried out unless otherwise specified. Results are presented as mean ± standard deviation (Mean ± SD) by using Microsoft Excel. Value of *p* < 0.05 was considered statistically significant. Calibration curves of the standards were considered as linear if *R*^2^ > 0.99. The comparison among all chemical methods used to investigate antioxidant activity was used to determine relative antioxidant capacity index (RACI). Pearson’s correlation coefficient was used to determine the relation between the variables by using Microsoft Excel. For more detailed insight in the relations between the variables, results were submitted to multivariate principal component analysis (PCA) using XLSTAT Version 2015.1 (Addinsoft Inc., New York, NY, USA).

## 4. Conclusions

This study supports the traditional use of *S. sonchifolius* to treat several ailments. Experimental studies of methanol extract of leaf tissue exhibited considerable antidiabetic and antioxidant activities. Significant differences between individual landraces were found and the results of this study showed that yacon could have a possible application in the pharmaceutical and nutraceutical fields, being a rich source of many bioactive compounds. The selection and propagation of specific landraces, supported by appropriate extraction procedure, would ensure greater concentration of active components and, consequently, a higher biological activity.

Our approach used some assays regarding reactive species and enzymes with biological significance (e.g., nitric oxide and superoxide anion radical scavenging activity and the cholinesterase inhibition), and results are reported here for the first time. Moreover, our HPLC protocol allowed the identification of 1,5-di-*O-*caffeoylquinic acid, which was not previously reported in yacon leaves.

The specific compounds responsible for yacon biological activities need to be found and further investigations for the most active compounds will be done in the near future.
